# Genome-wide association study of breast cancer in Latinas identifies novel protective variants on 6q25

**DOI:** 10.1038/ncomms6260

**Published:** 2014-10-20

**Authors:** Laura Fejerman, Nasim Ahmadiyeh, Donglei Hu, Scott Huntsman, Kenneth B. Beckman, Jennifer L. Caswell, Karen Tsung, Esther M. John, Gabriela Torres-Mejia, Luis Carvajal-Carmona, María Magdalena Echeverry, Anna Marie D. Tuazon, Carolina Ramirez, COLUMBUS Consortium, Luis Carvajal-Carmona, María Magdalena Echeverry, Mabel Elena Bohórquez, Rodrigo Prieto, Ángel Criollo, Carolina Ramírez, Ana Patricia Estrada, John Jairo Suáres, Gilbert Mateus, Jorge Mario Castro, Yesid Sánchez, Raúl Murillo, Martha Lucia Serrano, Carolina Sanabria, Justo Germán Olaya, Fernando Bolaños, Alejandro Vélez, Jenny Andrea Carmona, Alejandro Vélez, Nancy Guerrero Rodríguez, Cristina Serón Sousa, Cesar Eduardo Alvarez Mendez, Ana Isabel Orduz Galviz, Christopher R. Gignoux, Celeste Eng, Esteban Gonzalez-Burchard, Brian Henderson, Loic Le Marchand, Charles Kooperberg, Lifang Hou, Ilir Agalliu, Peter Kraft, Sara Lindström, Eliseo J. Perez-Stable, Christopher A. Haiman, Elad Ziv

**Affiliations:** 1 Division of General Internal Medicine, Department of Medicine, Institute of Human Genetics, University of California San Fancisco, San Francisco, California 94158, USA; 2 Department of Surgery, University of California San Francisco, San Francisco, California 94122, USA; 3 University of Minnesota Genomics Center, Minneapolis, Minnesota 55455, USA; 4 Cancer Prevention Institute of California, Fremont, California 94538, USA; 5 Division of Epidemiology, Department of Health Research and Policy, Stanford Cancer Institute, Stanford University School of Medicine, Palo Alto, California 94305, USA; 6 National Institute of Public Health, Cuernavaca 62100, Mexico; 7 Department of Biochemistry and Molecular Medicine, University of California Davis, Davis, California 95616, USA; 8 Research Group, Citogenética Filogenia y Evolución de Poblaciones, University of Tolima, Ibagué 7652000, Colombia; 9 Department of Genetics, Stanford University, Palo Alto, California 94305, USA; 10 Division of Pulmonary and Critical Care, Department of Medicine, Institute for Human Genetics, University of California San Francisco, San Francisco, California 94158, USA; 11 Department of Preventive Medicine, Norris Comprehensive Cancer Center, Keck School of Medicine, University of Southern California, Los Angeles, California 90033, USA; 12 University of Hawaii Cancer Center, Honolulu, Hawaii 96813, USA; 13 Division of Public Health Sciences, Fred Hutchinson Cancer Research Center, Seattle, Washington 98109, USA; 14 Department of Preventive Medicine, Robert H. Lurie Comprehensive Cancer Center, Feinberg School of Medicine, Northwestern University, Chicago, Illinois 60611, USA; 15 Department of Epidemiology and Population Health, Albert Einstein College of Medicine, Bronx, New York 10461, USA; 16 Program in Genetic Epidemiology and Statistical Genetics, Department of Epidemiology, Harvard School of Public Health, Boston, Massachusetts 02115, USA; 18 University of Tolima, Ibagué 7652000, Colombia; 19 Hospital Federico Lleras Acosta, Ibagué 7652000, Colombia; 20 Instituto Nacional de Canceroloǵia, Bogotá 11001000, Colombia; 21 Hospital Hernando Moncaleano, Neiva 41001, Colombia; 22 Dinámica IPS, Medellín 05001000, Colombia; 23 Hospital Pablo Tobon Uribe, Medellín 05001000, Colombia; 24 Universidad de Nariño, Pasto 52001, Colombia; 25 Clínica FOSCAL, Bucaramanga 68001, Colombia.

## Abstract

The genetic contributions to breast cancer development among Latinas are not well understood. Here we carry out a genome-wide association study of breast cancer in Latinas and identify a genome-wide significant risk variant, located 5′ of the Estrogen Receptor 1 gene (*ESR1*; 6q25 region). The minor allele for this variant is strongly protective (rs140068132: odds ratio (OR) 0.60, 95% confidence interval (CI) 0.53–0.67, *P*=9 × 10^−18^), originates from Indigenous Americans and is uncorrelated with previously reported risk variants at 6q25. The association is stronger for oestrogen receptor-negative disease (OR 0.34, 95% CI 0.21–0.54) than oestrogen receptor-positive disease (OR 0.63, 95% CI 0.49–0.80; *P* heterogeneity=0.01) and is also associated with mammographic breast density, a strong risk factor for breast cancer (*P*=0.001). rs140068132 is located within several transcription factor-binding sites and electrophoretic mobility shift assays with MCF-7 nuclear protein demonstrate differential binding of the G/A alleles at this locus. These results highlight the importance of conducting research in diverse populations.

## Abstract (web summary)

Genome-wide association studies (GWAS) have revealed gene variants associated with breast cancer, but their association with breast cancer development in Latinas is not clear. Here, the authors carry out a GWAS of breast cancer in Latinas and identify a significant protective variant of Indigenous American origin in the 6q25 region.

## Graphical abstract

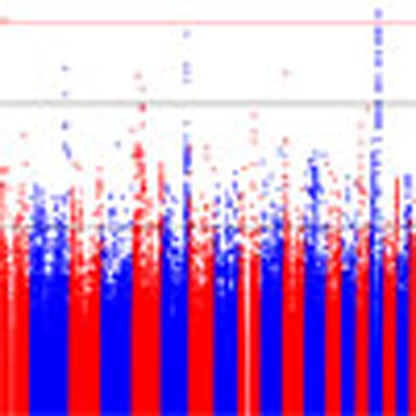

Breast cancer incidence varies substantially across racial and ethnic groups in the United States. The age-adjusted incidence of breast cancer in US European Americans and African Americans is 133.4 and 121.4 (per 100,000), respectively, whereas US women of Latin American origin have an incidence of 90.8 (ref. 1). Among Latina women, those with a high proportion of Indigenous American ancestry are at a lower risk of developing breast cancer^2^,^3^. This association could result from the correlation between genetic ancestry and environmental risk factors^4^, as well as genetics^5^. We have previously used admixture mapping for breast cancer in Latinas and identified 6q25 as a locus at which ancestry is associated with breast cancer^5^. Indigenous American ancestry at 6q25 is associated with protection from breast cancer, suggesting the association with global ancestry, could, at least in part, be because of genetic variants at that locus.

Since 2007, breast cancer genome-wide association studies (GWAS) have identified ~80 loci with small to moderate effects on risk (odds ratios (ORs) ranging from 1.05 go 1.53)^6^,^7^,^8^,^9^,^10^,^11^,^12^,^13^,^14^,^15^,^16^,^17^,^18^,^19^,^20^,^21^,^22^,^23^,^24^,^25^,^26^,^27^,^28^, with the vast majority of these studies limited to women of European ancestry, although GWAS has also been applied to populations of Asian^20^,^25^,^26^,^27^,^28^ and African ancestry^10^,^11^. GWAS of breast cancer in Latinas have not yet been published and may lead to the discovery of new disease-associated variants and to improved understanding of the genetic basis for health disparities, as recently demonstrated for type 2 diabetes^29^. Here we use a GWAS approach to find breast cancer risk variants in Latinas using data from two studies^2^,^5^. We report the discovery of a genome-wide significant breast cancer-protective variant of Indigenous American origin (rs140068132), located 5′ of the Estrogen Receptor 1 gene (*ESR1*; 6q25 region).

## Results

### Discovery GWAS and replication

We initially performed a GWAS of Latina women from the San Francisco Bay Area including 977 cases and 722 controls, from the San Francisco Bay Area Breast Cancer Study (SFBCS), the Northern California site of the Breast Cancer Family Registry (NC-BCFR) and the Genetics of Asthma in Latino Americans (GALA1, controls). We also conducted a GWAS of 520 cases and 2,491 controls from the Multiethnic Cohort (MEC). After imputation, the analysis of the San Francisco study, which used an Affymetrix 6.0 array, included 7,402,011 markers and the MEC, which used a combination of Illumina 1 and 2.5 M arrays, included 9,031,188 markers.

**Figure 1:**
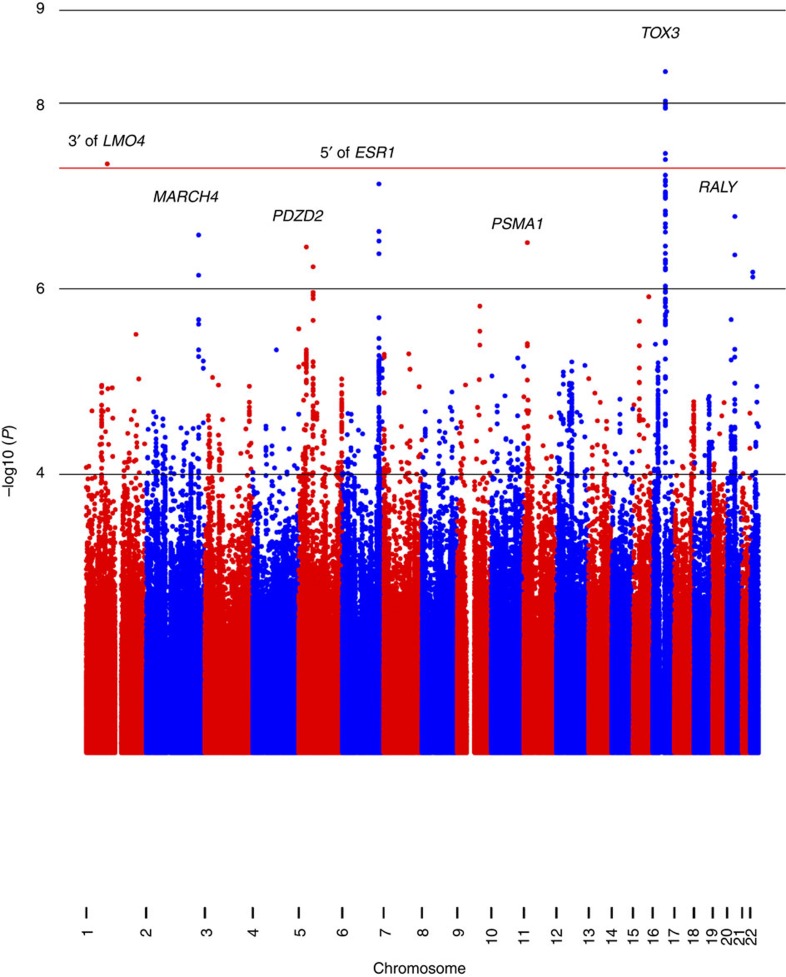
Manhattan plot of GWAS for breast cancer in 1,497 Latina cases and 3,213 controls. On the *x* axis are genomic positions by chromosome. On the *y* axis are the negative log_10_ *P* values for the association between the genetic variants and breast cancer risk. *LMO4*, LIM domain only 4; *MARCH4*, membrane-associated ring finger (C3HC4) 4, E3 ubiquitin protein ligase; *PDZD2*, PDZ domain containing 2; *ESR1*, oestrogen receptor 1; *PSMA1*, proteasome (prosome, macropain) subunit, alpha type, 1; *TOX3*, TOX high-mobility group box family member 3; *RALY*, RALY heterogeneous nuclear ribonucleoprotein.

**Figure 2:**
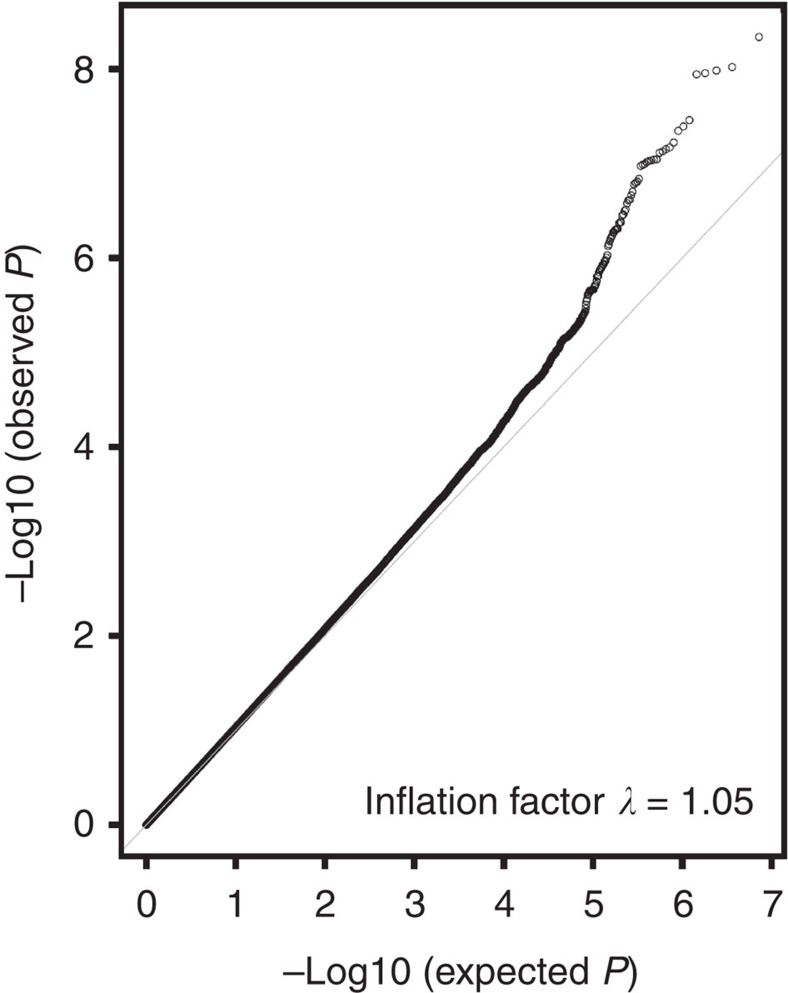
Quantile–quantile plot for GWAS of breast cancer in Latinas. The gray line represents a perfect match between the expected distribution of –log10 *P* under the uniform and those observed in the present analysis.

We performed a meta-analysis of 7,229,558 markers that overlapped between both data sets. In this meta-analysis that included 1,497 cases and 3,213 controls, only variant rs4784227, located at 3′ of the *TOX3* gene, a previously reported breast cancer risk locus at 16q12.1 (ref. 28), achieved genome-wide significance (*P*<5 × 10^−8^; T allele OR: 1.38 (95% CI: 1.24–1.54), *P*=3 × 10^−9^; *P* heterogeneity=0.24). The strongest associated variant outside the *TOX3* region was variant rs147157845, located ~50 kb upstream of the *ESR1* gene within a second known breast cancer risk locus at 6q25 (OR: 0.59 (95% CI: 0.50–0.71), *P*=1.5 × 10^−7^; *P* heterogeneity=0.47; imputation information co-efficient=0.92; Fig. 1). The quantile–quantile (Q–Q) plot and the corresponding genomic inflation factor showed that adjustment by the first 10 principal components dealt appropriately with issues of population structure (*λ*=1.05; Fig. 2).

We selected 14 single-nucleotide polymorphisms (SNPs) with *P*<9 × 10^−7^ for replication testing in an independent sample of 977 cases and 1,158 controls from a population-based breast cancer study in Mexico^30^, of which nine were successfully genotyped using Sequenom iPLEX assays. Two SNPs located within the 6q25 region (rs147157845 and rs140068132) showed highly statistically significant associations with effect sizes similar to those in the discovery phase (Table 1). None of the other SNPs were associated with breast cancer risk (*P*<0.05) in the replication sample (Supplementary Table 1). Variants rs147157845 (Chr6: position 151971376, allele frequencies in US Latinas: C: 0.91, A: 0.09) and rs140068132 (Chr6: position 151954834, allele frequencies: A: 0.91, G: 0.09) are 16.5 kb apart and are in strong linkage disequilibrium (LD; *r*^2^=0.97). For further validation, we tested the association of the rs140068132/rs147157845 SNPs with breast cancer risk in a sample of 546 Colombian women with breast cancer and 440 Colombian women without breast cancer, which also showed a strong association, and in Latinas from the Women’s Health Initiative (WHI) SHARe Study (120 cases and 3,373 controls), which replicated both the magnitude and direction of the associations (Table 1).

### Fine mapping at 6q25 region and association with ancestry

**Figure 3:**
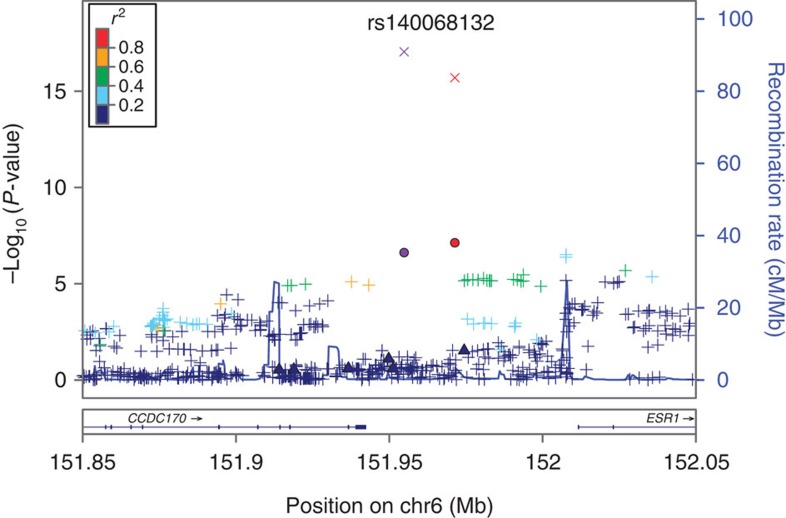
A regional plot of the −log10 *P* values for SNPs at 6q25.1. The SNP with the highest −log10 *P* value is coloured purple and identified by its rs no. on top of the graph. The two SNPs that were replicated in multiple independent samples are circle-shaped, updated −log10 *P* values after meta-analysis are X-shaped, and previously reported risk SNPs are triangle-shaped. All other SNPs are represented by crosses and the colours reflect the level of correlation with the SNP with highest −log10 *P* value. The LD is estimated using data from 1,000 Genomes Project Amerindian populations. In addition, shown are the SNP Build 37 coordinates in megabases (Mb), recombination rates in centimorgans (cM) per megabase (Mb) and the name and location of genes in the UCSC Genome Browser (below).

The rs140068132/rs147157845 SNPs are relatively common in Latin American populations (5% in Puerto Ricans, 10% in Colombians and 14% in Mexicans from the 1KGP) but are absent in Europeans and Africans, and they have a very low frequency in East Asians (Supplementary Table 2). Neither these variants nor any correlated SNPs in Latinos were found to be in LD with the index variants in the region previously reported in Asians and Europeans^12^,^18^,^31^ (Fig. 3; Supplementary Fig. 1). In addition, none of the previously reported variants at 6q25 showed genome-wide statistically significant associations in the Latinas (Supplementary Table 3). One GWAS-identified variant at 6q25 (rs9383938) had an associated *P* value of 0.03 in the Latinas and the direction of the OR was concordant with that previously reported 7. In a model that included rs140068132 and rs9383938, the association between breast cancer and rs140068132 was not attenuated (OR 0.60; 95% CI 0.49–0.73).

We sequenced a 245-K region (positions 15,176,132 to 152,010,131) at 6q25 in 451 cases and 456 controls from the MEC and did not identify any new variants (with minor allele frequency >1%) that were associated with breast cancer. We did find three variants (rs4524618, rs9397432 and rs79692348) that had more significant *P* values in the sequencing data (Supplementary Table 4). However, when those same SNPs were analysed in the full data set, they were less strongly associated with breast cancer (*P* values 1.23 × 10^−5^, 1.25 × 10^−5^, and 7.93 × 10^−6^). Furthermore, in conditional analyses with the full data set, they were all nonsignificant in models that included rs140068132. In a previous admixture-mapping analysis that included a subset of these samples, higher Indigenous American ancestry within the 6q25 locus was associated with lower risk of breast cancer^5^. Since the minor alleles of rs140068132 and rs147157845 are protective and are only common in Latinos, we reasoned that they are likely common in Latinos because of Indigenous American ancestry. We checked the allele frequency of rs140068132 in women for whom the locus-specific ancestry at 6q25 was estimated to be homozygous Indigenous American and found the allele frequency to be 25% among controls. In contrast, women for whom the locus-specific ancestry at 6q25 was estimated to be homozygous European had an allele frequency of 0.1%. Next, we used multivariate logistic regression to determine whether the association with locus-specific ancestry at 6q25 is attenuated after adjusting for the rs140068132 genotype. We found that after adjusting for rs140068132, the magnitude of the log OR for local ancestry was reduced by 35% (Table 2).

### Subphenotype analyses

In analyses by oestrogen receptor (ER) status, the associations with variants rs147157845 and rs140068132 were stronger for ER− disease versus ER+ disease (*P* heterogeneity=0.01; Table 3). The same trend was observed in Colombians, for whom ER status was available for a subset of cases (Supplementary Table 5).

**Figure 4:**
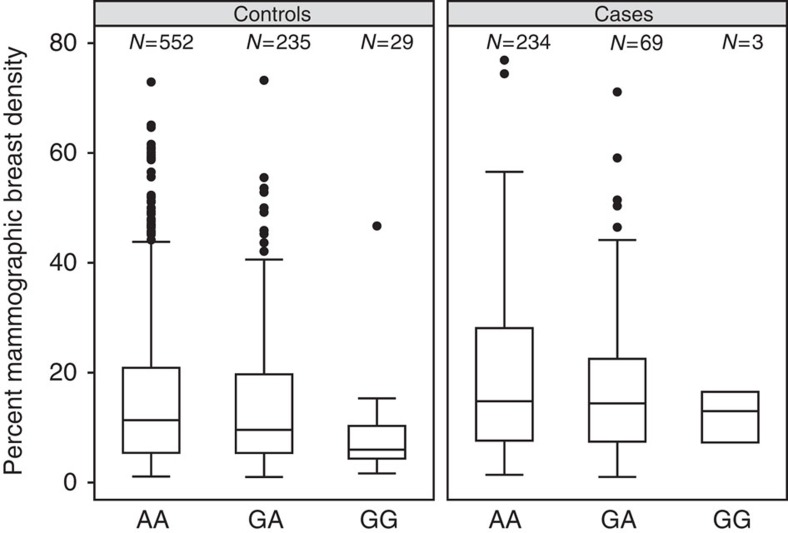
Box plot of percent mammographic breast density by genotypes for SNP rs140068132 at 6q25 in 1,113 women (304 cases and 809 controls) from the Mexican study. The boxes represent the median (black middle line) limited by the 25th (Q1) and 75th (Q3) percentiles. The whiskers are the upper and lower adjacent values, which are the most extreme values within Q3+1.5(Q3−Q1) and Q1−1.5(Q3−Q1), respectively. The black dots represent outliers. *N* defines the number of individuals within each genotype category.

We investigated the association between the rs140068132 SNP and mammographic density in 1,113 women (304 cases and 809 controls) from the Mexican study for whom mammographic density measures were available. In multivariate models, we found a significant association between mammographic density and the variants at 6q25 among the 809 controls (rs140068132 *P*=0.001; rs147157845 *P*=0.005). The mean density among control women homozygous for the rs140068132 common allele (AA) was 16% (95% CI: 14–17%; *N*=549), while among women homozygous for the protective allele (GG) the mean density was 8% (95% CI: 5–12%; *N*=29; Fig. 4). After adjusting for mammographic breast density, the magnitude of the log OR for the rs140068132 polymorphism on breast cancer risk was reduced by 16% (Supplementary Table 6).

### Replication of previously reported associations

**Figure 5:**
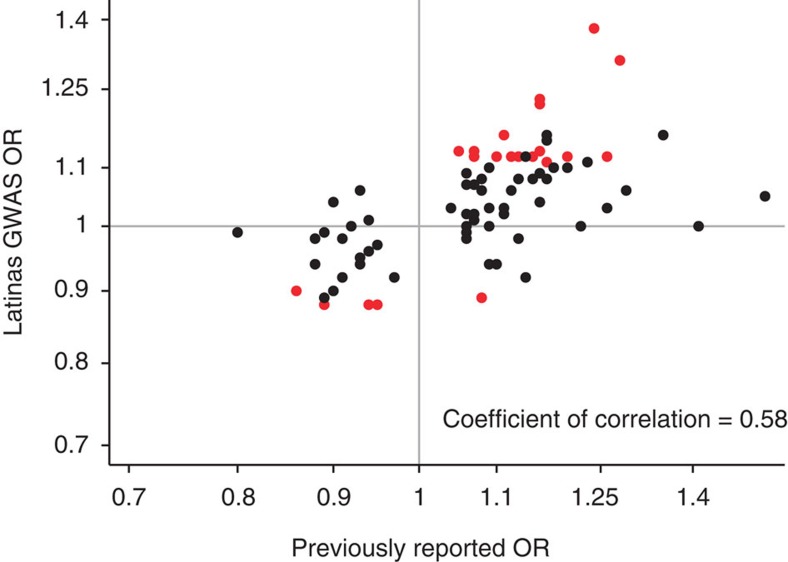
Replication of previously reported associations in Latinas. Scatter plot of odds ratios previously published (*x* axis) and in Latinas (*y* axis). Red dots represent SNPs that were associated at ≤0.05 level of significance in Latinas.

We also confirmed the association with many of the risk variants previously reported for breast cancer risk (Fig. 5). The allele frequencies for these variants in Latinas were strongly correlated with frequencies reported for European women (Supplementary Fig. 2). Twenty-three of the eighty-three previously reported variants were associated at *P*<0.05, and the majority of SNPs (84%) had ORs that were directionally concordant with those previously reported (Supplementary Table 3).

### EMSA for rs140068132 alleles

We found evidence that rs140068132 may be a functionally relevant variant. Rs140068132 falls within the sequence of several transcription factor-binding sites (TFBS) in ENCODE data including *FOXA1*, *p300*, *CTCF*, *HA-E2F1*, *GATA3* and *FOXA2*, most of which have been implicated in ER function and/or breast cancer^32^,^33^,^34^,^35^,^36^ (Supplementary Fig. 3). Furthermore, MAPPER^37^ predicted the SNP to be critical to at least four putative TFBS, three of which were disrupted with the G versus A allele at rs140068132. To test functional relevance, two 25-mer biotinylated probes of identical sequence except for the A/G alleles at position chr6:151954834 were synthesized (IDT DNA, chr6:151954823-151954847). These were designed to encompass the four putative TFBS predicted by MAPPER. Electrophoretic mobility shift assays (EMSA) were then performed interrogating binding of the 25-mer probes to nuclear protein extracts derived from MCF-7 cells, and they demonstrated the differential binding of probes with the A versus G allele, with far less binding of the probe containing the protective G allele (Supplementary Fig. 4). Furthermore, competition with an identical but unbiotinylated probe at 500-fold concentration demonstrated specificity of the dominant-binding product.

## Discussion

We performed a GWAS for breast cancer in Latinas and identified an association between two linked variants within the 6q25 region, 5′ of the *ESR1* gene and breast cancer risk. The minor allele is protective: the odds of having breast cancer decrease by a factor of 0.60 per allele. We also found that the minor allele is more protective against ER-negative disease and that it is associated with decreased mammographic density. The rs140068132/rs147157845 SNPs are relatively common in Latin American populations, ranging between 5 and 15% depending on the proportion of Indigenous American ancestry of the region but are rare (<2%) or absent in other groups. These variants were not in LD with the index variants reported in Asians and Europeans, which suggests that the newly discovered SNPs represent a novel signal that is specific to populations with Indigenous American ancestry.

Previous studies have reported some risk variants at 6q25 to be more strongly associated with ER-negative disease^7^,^20^,^38^. Our findings also showed different association effect sizes by ER status, with the rare variant at rs140068132 being more protective for ER-negative disease. Further work will be necessary to understand the biological mechanism behind this difference.

We have previously conducted a breast cancer admixture-mapping analysis, which showed that higher Indigenous American ancestry within the 6q25 locus was associated with lower risk of breast cancer^5^. Adjusting for rs140068132 reduced the magnitude of the association with local ancestry but did not eliminate it completely. The residual locus-specific ancestry association suggests that there may be additional rare ancestry-informative variants associated with breast cancer at this locus that the present study might not have detected.

High mammographic density is one of the strongest risk factors for breast cancer. Some of the SNPs associated with breast cancer risk have also been associated with breast density^39^,^40^,^41^,^42^, which suggests that breast density may mediate genetic associations. We showed that the rs140068132-protective variant was associated with lower mammographic density and after adjusting for mammographic breast density, the magnitude of the log OR for the rs140068132 polymorphism on breast cancer risk was partially reduced. This suggests that the SNP might affect breast cancer risk both through and independently of its effect on breast density.

We used both sequencing and *in vitro* assays (EMSA) to help fine map and assess functionality. The sequence data did not identify any novel SNP in the region that could explain the association. Given that the rs140068132 SNP is located within the binding site of multiple transcription factors, we conducted EMSA assays to evaluate whether the two alleles at this position had differential binding affinity to nuclear protein. Results clearly showed that the protective G allele had a reduced binding affinity to nuclear protein compared with the A allele. While our work suggests that the A/G SNP at rs140068132 has functional relevance, further work will be necessary to understand the exact mechanism by which rs140068132 confers decreased breast cancer risk.

In summary, in this GWAS of breast cancer in Latinas we report a risk variant at the 6q25 locus that, to our knowledge, represents the strongest association effect size on breast cancer for a common variant reported to date. The rs140068132 variant is limited to populations of Indigenous American ancestry, explained at least part of the previously reported association between breast cancer risk and genetic ancestry at the 6q25 locus in Latinas and may partially account for the lower incidence of breast cancer in this population. Overall, our findings highlight the importance of conducting genetic studies in non-European ancestry populations. Additional larger efforts in minority populations may reveal novel variants and loci contributing to complex traits.

## Methods

### Human samples

All participants provided written informed consent and the studies were approved by local Human Subjects Committees: The Fred Hutchinson Cancer Research Center IRB (Institutional Review Board), Comité de Ética en Investigación (CEI), Ethics Committee of the University of Tolima, Cancer Prevention Institute of California IRB, University of Southern California IRB and the University of California San Francisco IRB.

The following studies provided samples for the discovery GWAS:

SFBCS: The SFBCS is a population-based multiethnic case–control study of breast cancer^43^,^44^. Cases aged 35–79 years diagnosed with invasive breast cancer from 1995 to2002 were identified through the Greater Bay Area Cancer Registry. Controls were identified by random-digit dialing and matched on 5-year age groups. Blood collection was initiated in 1999. The present analysis includes 351 cases and 579 controls from this study who self-identified as Latina or Hispanic.

BCFR: The BCFR is an international, National Cancer Institute (NCI)-funded family study that has recruited and followed over 13,000 breast cancer families^45^. The present study includes samples from the population-based NC-BCFR. Cases aged 18–64 years diagnosed from 1995 to 2007 were ascertained through the Greater Bay Area Cancer Registry. Cases with indicators of increased genetic susceptibility (diagnosis at the age of <35 years, bilateral breast cancer with the first diagnosis at the age of <50 years, a personal history of ovarian or childhood cancer and a family history of breast or ovarian cancer in first-degree relatives) were oversampled. Cases not meeting these criteria were randomly sampled^46^. Population controls were identified through random-digit dialing and frequency-matched on 5-year age groups to cases diagnosed from 1995 to 1998. We included 641 cases and 61 controls who self-identified as Latina or Hispanic from this study.

MEC: MEC is a large prospective cohort study in California (mainly Los Angeles County) and Hawaii^47^. The breast cancer study is a nested case–control study including women with invasive breast cancer diagnosed at the age of >50 years and controls matched on age and self-identified ethnicity^47^. For the current study, we used data and DNA samples from 546 Latina women with breast cancer and 558 matched Latina controls. We also included an additional 1,941 controls who self-identified as Hispanic/Latino from this study (935 of these controls are men).

GALA1: GALA1 is a family-based study (including children with asthma and their parents) of pediatric asthma in Latino Americans^48^. The sample includes 294 individuals of Mexican origin and 365 individuals from Puerto Rico. We included 112 females of self-reported Mexican origin from the GALA1 study to our set of population controls. The individuals are between 11 and 42 years of age (85% are older than 20 years).

In order to replicate the top associations from the discovery GWAS, we analysed multiple independent samples from the following studies:

The Mexico Breast Cancer Study: This study is a population-based case–control study of breast cancer conducted in Mexico City, Monterrey and Veracruz^30^. Cases aged 35–69 years diagnosed between 2005 and 2007 were recruited from 11 hospitals (three to five in each region). Controls were recruited based on membership in the same health plan as the cases and are frequency-matched on 5-year age groups. For the current study, we used data and DNA samples from 977 women with breast cancer and 1,158 controls.

Colombian Study of Environmental and Heritable Causes of Breast Cancer (COLUMBUS): COLUMBUS is a population-based case–control study of breast cancer conducted in four cities: Bogota, Ibague and Neiva, from the Central Colombian Andes region, and Pasto, from the Colombian South. Incident cases with invasive breast cancer aged 18–75 years have been recruited in two population registries and two large cancer hospitals. Recruitment started in 2011. Cancer-free controls were recruited through the same institutions and were matched on education, socioeconomic status and local origin using a genealogical interview. In the current study, we genotyped DNA samples from 546 cases and 440 controls.

WHI SNP Health Association Resource (WHI SHARe): WHI is a long-term national health study that focuses on understanding risk factors for common diseases such as heart disease, cancer and fractures in postmenopausal women. A total of 161,838 women aged 50–79 years were recruited from 40 clinical centres in the United States between 1993 and 1998 (refs 49, 50). Medical history was updated at least annually. Breast cancers were verified by medical records and pathology report review^51^,^52^. The WHI SHARe includes 3,493 self-identified Hispanic/Latina women from WHI who provided consent for DNA analysis and were genotyped on the Affymetrix 6.0 array. WHI SHARe was imputed to the 1000 Genomes Project using MACH^53^. Breast cancer cases were defined as cases with incident invasive breast cancer. We included 120 cases and 3,373 controls from this study.

### Genotyping and quality-control procedures

The SFBCS, NC-BCFR and GALA1 samples were genotyped with the Affymetrix 6.0 array according to the manufacturer’s instructions in the Laboratory of Esteban Gonzalez Burchard at UCSF. The MEC samples were genotyped with the Illumina Infinitum 660 W-Quad or the Omni 2.5 array in the Genomics Center at USC and at the Broad Institute (Cambridge, MA, USA).

Before imputation, we excluded 15 cases and 30 controls from the SFBCS/NC-BCFR/GALA1 set that had a genotyping call rate <95% or showed either known or cryptic relatedness. We excluded 26 cases and 8 controls from the MEC because of unexpected relatedness. The final analysis included 1,497 cases and 3,213 controls (1,699 individuals from the SFBCS/NC-BCFR/GALA1 set (977 cases and 722 controls) and 3,011 from the MEC (520 cases and 2,491 controls)).

A scatter plot of the first and second principal components estimated for the US Latina samples included in the discovery phase of the study showed the expected distribution for this population, with most samples spreading between the European and Asian axes (and beyond the Asian cluster towards what would be the Indigenous American cluster) and a smaller proportion of samples deviating towards the African cluster (Supplementary Fig. 5).

### Imputation

The discovery GWAS included genotyped as well as imputed SNPs for better coverage. Sample genotypes were phased and missing markers were imputed using the software IMPUTE2 (ref. 54). Phased data of 1,094 samples from the 1000 Genomes Project (www.1000genomes.org) were used as the reference data set. These samples are from African, African American, Asian, Caucasian and Native American populations. We excluded SNPs with an information coefficient value <0.5 and minor allele frequency <1%. For markers in the MEC data, we conducted additional stringent quality-control analyses to exclude SNPs with different minor allele frequencies in controls typed on or imputed from different genotyping platforms. SNPs that exhibited differences in minor allele frequencies between the controls genotyped with the Illumina Infinitum 660 W-Quad or Omni 2.5 M platforms at *P*<0.01, and/or had ORs >10 or <0.1 were excluded. The final GWAS analysis included 7,229,558 SNPs.

### SNP and genotyping for replication in Mexican women

SNPs were selected for replication in an independent sample of Mexican women according to the following criteria: (1) the polymorphic site was an SNP or captured by an SNP, as opposed to insertion/deletion; (2) *P* value <5 × 10^−7^; (3) SNPs that have not been previously associated with breast cancer risk; and (4) when multiple SNPs were in high LD (*r*^2^>0.8), one or two with the lowest *P* value were selected for genotyping.

A total of 14 SNPs were selected for replication. Four SNPs failed during assay design. Genotyping for the replication of 10 SNPs was completed using Sequenom iPLEX assays run on the MassArray Analyzer 4 platform at the University of Minnesota Genomics Center. One SNP failed the experimental assay and was excluded from the analysis. Each sample plate included one to three duplicates with a replication genotyping concordance of 100%. Two SNPs had a call rate of 99.7% and the remaining seven SNPs had a call rate of 100%. We excluded all individuals with any missing genotypes from further analysis (six cases and six controls). The final analysis included 977 cases and 1,158 controls. Genetic ancestry was estimated for all Mexican samples using a previously described panel of 106 ancestry informative markers^3^. These markers were genotyped with a iPLEX Sequenom assay^3^. Ancestry estimation was performed with the FRAPPE^55^ and STRUCTURE^56^ programmes including ancestral genotypes as previously described^2^.

### Genotyping of rs140068132/rs147157845 in Colombians

Samples from COLUMBUS were genotyped for rs140068132 and rs14757845 using the KASP genotyping technology following the manufacturer’s instructions. Samples were genotyped in 384-well plates that contained positive genotyping controls for the three genotypes and two duplicates. The call rates for rs140068132 and rs14757845 were 98.0% and 98.2%, respectively. The final analyses included 546 cases and 440 controls. Association testing and meta-analyses were carried out with PLINK^57^.

### Sequencing of 245 K region in MEC study samples

We sequenced the ESR1 region at 6q25 in 451 breast cancer cases and 456 controls from MEC as part of a multiethnic targeted sequencing project of known breast cancer GWAS loci. Region boundaries were defined by nearest recombination hotspot downstream and upstream from the original GWAS signal (rs2046210) as identified using the HapMap CEU population. Briefly, 12 GWAS-identified regions covering 5,500 kb were hybrid-captured and sequenced. Sequencing was performed at the Broad Institute and details of the sequencing method have been described elsewhere^58^. We were not able to capture 2,740 kb (48.9%) of the originally targeted sequence primarily because of repetitive sequence content. The median proportion of captured regions with coverage >20 × was higher than 93% across all regions (range 93.8–99.9). We used Burrows-Wheeler Aligner (BWA)^59^ to align reads to the genome and GATK^60^,^61^ with default standard filters for genotype calling.

The sequence data at 6q25 encompassed 245 K (positions: 151765132 to 152010131). Of a total of 4,936 variants observed, 443 had a minor allele frequency of >0.01.

### Statistical analysis

We used logistic regression models to estimate the association between SNPs and breast cancer risk. ORs were estimated for the variant allele based on a log-additive model and adjusted for the first 10 principal components. PLINK version 1.06 (ref. 57) was used to analyse genome-wide data. The SFBCS, NC-BCFR and GALA1 samples were pooled as one data set. The MEC samples were analysed as a separate data set. Meta-analysis of the two data sets was performed using the METAL package, which performs an inverse-variance-weighted combination of the association effect sizes for each study and provides *P* values for heterogeneity.

In order to use a systematic approach to analyse the replication of previously reported associated variants, we decided to only include previously published SNPs that were registered in the Catalog of Published Genome-Wide Association Studies compiled by the National Human Genome Research Institute. In addition, we used a restriction criteria of *P* value lower than 5 × 10^−8^.

Replication analyses of newly discovered variants in Mexican women, Colombian women and in Latinas from WHI were conducted using logistic regression models. The analysis in Mexicans was adjusted for age at diagnosis and global genetic ancestry. For the WHI samples, genotypes for the rs140068132/rs147157845 SNPs were available from genome-wide genotypes that were generated as part of the WHI SHARe GWAS. The WHI analysis included adjustment for principal components 1 to 4, region and age at diagnosis. The Colombian samples included women from two different regions (Central and South Colombia) who were analysed separately and were combined in a meta-analysis using the inverse-variance method. The final meta-analysis with all studies was conducted using the same method.

Genome-wide association analyses were performed for cases versus controls as well as for ER-negative (ER−) and ER-positive (ER+) cases versus controls. Among the MEC cases with available ER status, 303 were ER+ and 108 ER−. Of the SFBCS/NC-BCFR cases, 505 were ER+ and 185 ER−. The analyses by hormone receptor status were based on 808 ER+ and 293 ER− cases (303 ER+ and 108 ER− from MEC and 505 ER+ and 185 ER− from SFBCS/NC-BCFR).

We used logistic regression to evaluate the effect of global and locus-specific ancestry on breast cancer risk when the SNPs rs140068132/rs147157845 were included in the model. We also included the first 10 principal components, age and study as covariates.

We used linear regression to evaluate the association between the rs140068132/rs147157845 polymorphisms and percent breast density among controls from the Mexican study. Percent mammographic density was log-transformed in order to obtain a distribution that would more closely approximate normality. We included age, body mass index, height, parity, breastfeeding, Indigenous American ancestry and African ancestry as covariates. These analyses were conducted with the STATA 12.1 software^62^.

ENCODE: The UCSC genome browser (genome.ucsc.edu) was used to get an overview of the genetic architecture surrounding our SNPs of interest, and specifically whether the SNPs fell within TFBSs determined by ENCODE ChIP-seq data.

MAPPER^37^: MAPPER was used at default settings to interrogate *Homo sapiens* sequence and bioinformatically predict putative transcription factor sites to which our SNP was critical. We took significant putative sites to be *E* value <15.

### Percent mammographic density measure

Percent mammographic density measures and potential confounder variables (age at diagnosis or interview, height, body mass index, parity and breastfeeding) were available for 1,113 (304 cases and 809 controls) women from the Mexican study. Information on covariates was obtained by questionnaire. Mammograms from cases were collected from participating hospitals; those from controls were performed at the same hospitals. Craniocaudal views were digitized using an Astra 2400S scanner (Umax, Fremont, CA, USA). A single observer measured mammographic density using Mamgr, a computer-assisted programme developed at the Department of Epidemiology and Population Health, London School of Hygiene and Tropical Medicine^63^. For cases, the contralateral craniocaudal view was measured, and for controls the left craniocaudal view was measured. Percent breast density was calculated by dividing the dense area by the total breast area. The Mamgr observer was blinded to questionnaire data. Mammographic density measurements using the Mamgr have been compared with the Cumulus programme developed at the University of Toronto, with a reported intraclass correlation of 0.87 (ref. 64).

### Electrophoretic mobility shift assay

MCF-7 cells (ATCC) were grown in DMEM high-glucose media with 10% fetal bovine serum in T25 flasks using standard cell culture practices. Nuclear protein extracts were harvested from passage 2 cells at 75% confluence and passage 3 cells at 85% confluence using the Pierce Scientific’s NE-PER nuclear and cytoplasmic extract kit with addition of Pierce Halt protease inhibitor cocktail. Biotinylated probes were synthesized and high-performance liquid chromatography purified from IDT DNA. EMSA were performed using LightShift Chemiluminescent EMSA kit (Pierce Scientific). The control reactions were performed as suggested by the protocol, except complete quenching of specific binding was not obtained at 200-fold concentration of unbiotinylated competitor but was obtained at 500-fold excess competitor. The kit protocol was followed with optimization to include the following components in each 20 μl reaction in the following order when relevant: water (variable), binding buffer 1X, poly dI.dC 50 ng μl^−1^, unbiotinylated primer 10 pmol, 2 μl nuclear protein extract and biotinylated primer 20 fmol. Preincubation of all components was performed on ice for 2 min before addition of the biotinylated primer, and all components were then incubated for 55 min at room temperature before running the gel. Polyacrylamide gel (6%) was pre-run for 50 min, run for 1 h 15 min after loading and transferred to membrane for 45 min. Thereafter, crosslinking and washes were performed as per protocol. The experiment was replicated four times, twice each with protein derived from two different passages of MCF-7 cells, with similar results.

## Additional information

**How to cite this article**: Fejerman, L. *et al*. Genome-wide association study of breast cancer in latinas identifies novel protective variants on 6q25. *Nat. Commun.* 5:5260 doi: 10.1038/ncomms6260 (2014).

## Supplementary Material

Supplementary Information Supplementary Figures 1-5 and Supplementary Tables 1-6

## Figures and Tables

**Table 1:** Discovery and replication of newly discovered protective variants at 6q25 related to breast cancer risk in Latinas.

***6q25*** **Region**	**Alleles** *	**OR**	**95% CI**	***P*** **value**	**MAF** †
*Discovery (1,497 cases/3,213 controls)*					
rs140068132	A/G	0.60	0.49–0.72	3 × 10^−7^	9%
rs147157845	C/A	0.59	0.48–0.72	1 × 10^−7^	9%
*Replication Mexicans (977 cases/1,158 controls)*					
rs140068132		0.63	0.53–0.75	3 × 10^−7^	15%
rs147157845		0.66	0.55–0.78	3 × 10^−6^	15%
*Replication Colombians (546 cases/440 controls)*					
rs140068132		0.54	0.41–0.71	1 × 10^−5^	10%
rs147157845		0.55	0.42–0.72	2 × 10^−5^	10%
*Replication WHI Hispanics (120 cases/3,373 controls)*					
rs140068132		0.61	0.31–1.22	0.16	7%
rs147157845		0.60	0.30–1.19	0.15	7%
*Meta-analysis (3,140 cases/8,184 controls)*					
rs140068132		0.60	0.53–0.67	9 × 10^−18^	
rs147157845		0.61	0.54–0.68	2 × 10^−16^	

CI, confidence interval; MAF, minor allele frequency; OR, odds ratio; WHI, Women’s Health Initiative.

^*^Reference allele/tested allele.

^†^MAF: tested allele.

**Table 2:** Association between breast cancer, global Indigenous American (IA) ancestry, local IA ancestry and rs140068132/rs147157845*.

	**OR**	**95% CI**	***P*** **value**
*Model 1*			
Global IA ancestry	0.31	0.18–0.54	3.47 × 10^−5^
*Model 2*			
Global IA ancestry	0.55	0.29–1.01	0.056
Local IA at 6q25	0.55	0.42–0.72	1.87 × 10^−5^
*Model 3*			
Global IA ancestry	0.55	0.30–1.02	0.06
Local IA at 6q25	0.68	0.50–0.91	0.009
rs140068132	0.63	0.50–0.80	1.6 × 10^−4^

CI, confidence interval; IA, Indigenous American; MEC, Multiethnic Cohort; NC-BCFR, Northern California site of the Breast Cancer Family Registry; OR, odds ratio; SFBCS, San Francisco Bay Area Breast Cancer Study.

^*^These analyses included 1,476 US Latina cases and 1,131 controls from the SFBCS, the NC-BCFR and the MEC with available locus specific ancestry estimates from a previous study.

**Table 3:** Association between rs140068132/rs147157845 and breast cancer risk by ER status in US Latinas.

**ER status (808 ER+, 293 ER−, 3,221 controls)**	**OR** *	**95% CI**	***P*** **value**
*rs140068132*			
ER+	0.63	0.49–0.80	1.9 × 10^−4^
ER−	0.34	0.21–0.54	4.7 × 10^−6^
ER− versus ER+	0.53	0.32–0.88	0.014
*rs147157845*			
ER+	0.62	0.48–0.79	9.3 × 10^−5^
ER−	0.34	0.21–0.54	3.8 × 10^−6^
ER− versus ER+	0.55	0.34–0.91	0.019

CI, confidence interval; ER, oestrogen receptor; ER+, oestrogen receptor-positive; ER−, oestrogen receptor-negative; OR, odds ratio.

^*^ORs are for the minor alleles (rs140068132 allele G and rs147157845 allele A).
