# Supplementary material for: Genome-wide association study of breast cancer in Latinas identifies novel protective variants on 6q25
Source: Nat Commun. 2014 Oct 20;5:5260. doi: 10.1038/ncomms6260 (PMC4204111; doi:10.1038/ncomms6260)

Table 1: Replication of 9 top hits from the Latinas discovery GWAS analysis in 977 Mexican breast cancer cases and 1158 controls

Chr.	rs_id #	Position	Alleles*	OR	95%CI	P-value
11	rs117641562	108543494	T/C	1.01	0.87 - 1.16	0.93
6	rs140068132	151954834	A/G	0.63	0.53 - 0.75	$3.5x10^{-7}$
6	rs147157845	151971376	C/A	0.66	0.55 - 0.78	$3.2x10^{-6}$
2	rs207809	217143742	G/T	1.05	0.89 - 1.24	0.54
20	rs60931694	17565525	G/T	1.27	0.88 - 1.84	0.21
5	rs717163	31857433	A/C	0.95	0.75 - 1.21	0.69
21	rs7278425	46926551	C/T	1.00	0.86 - 1.17	0.96
12	rs7311611	64221421	A/G	1.02	0.89 - 1.17	0.77
20	rs8119836	17551047	C/A	1.17	0.78 - 1.73	0.45

*Reference/Tested (Common/Minor)

Table 2: 1000 Genomes allele and genotype frequencies for rs140068132 SNP

Pop.	Alleles A	Alleles G	A|A	A|G	G|G	Allele count	Genotype count
ALL	0.982	0.018	0.965	0.034	0.001	2145(A)/39(G)	1054(A|A)/37(A|G)/1(G|G)
AFR	0.998	0.002	0.996	0.004		491(A)/1(G)	245(A|A)/1(A|G)
AMR	0.903	0.097	0.812	0.182	0.006	327(A)/35(G)	147(A|A)/33(A|G)/1(G|G)
ASN	0.995	0.005	0.99	0.01		569(A)/3(G)	283(A|A)/3(A|G)
ASW	0.992	0.008	0.984	0.016		121(A)/1(G)	60(A|A)/1(A|G)
CEU	1		1			170(A)	85(A|A)
CHB	0.985	0.015	0.969	0.031		191(A)/3(G)	94(A|A)/3(A|G)
CHS	1		1			200(A)	100(A|A)
CLM	0.9	0.1	0.8	0.2		108(A)/12(G)	48(A|A)/12(A|G)
EUR	1		1			758(A)	379(A|A)
FIN	1		1			186(A)	93(A|A)
GBR	1		1			178(A)	89(A|A)
IBS	1		1			28(A)	14(A|A)
JPT	1		1			178(A)	89(A|A)
LWK	1		1			194(A)	97(A|A)
MXL	0.864	0.136	0.742	0.242	0.015	114(A)/18(G)	49(A|A)/16(A|G)/1(G|G)
PUR	0.955	0.045	0.909	0.091		105(A)/5(G)	50(A|A)/5(A|G)
TSI	1		1			196(A)	98(A|A)
YRI	1		1			176(A)	88(A|A)

AFR: African; AMR: Amerindian; ASN: East Asian; ASW: African Americans from the U.S. Southwest; CEU: Utah residents with Northern and Western European ancestry; CHB: Han Chinese in Beijing, China; CHS: Southern Han Chinese, China; CLM: Colombian in Medellin, Colombia; EUR: European; FIN: Finnish in Finland; GBR: British in England and Scotland; IBS: Iberian populations in Spain; JPT: Japanese in Tokyo, Japan; LWK: Luhya in Webuye, Kenya; MXL: Mexican Ancestry in Los Angeles, California; PUR: Puerto Rican in Puerto Rico; TSI: Toscani in Italy; YRI: Yoruba in Ibadan, Nigeria.

Table 3: Breast cancer risk associations in U.S Latinas for previously reported variants, overall and by hormone receptor status

RS #	Chr.	Position	Alleles*	Freq. Latinas^	Freq. Pub^	OR Pub.	95% CI Pub.	OR Lat.	95% CI Lat.	P Lat.	OR ER-neg	P ER-neg	OR ER-pos	P ER-pos	Reference
rs616488	1	10566215	A/G	0.43	0.33	0.94	0.9-0.98	0.96	0.87-1.07	0.458	0.84	0.068	1	0.971	Michailidou K (2013)
rs11552449	1	114448389	C/T	0.35	0.17	1.08	1.02-1.14	0.89	0.8-0.99	0.031	0.93	0.428	0.88	0.059	Michailidou K (2013)
rs11249433	1	121280613	T/C	0.26	0.39	1.16	1.09-1.24	1.13	1-1.27	0.045	1.26	0.036	1.09	0.248	Thomas G. (2009)
rs6678914	1	202187176	G/A	0.29	0.41	1.1	1.06-1.13	0.94	0.85-1.05	0.305	0.81	0.037	1.01	0.842	Garcia Closas (2013)
rs4245739	1	204518842	C/A	0.74	0.74	1.14	1.1-1.18	0.92	0.83-1.03	0.164	0.83	0.058	1	0.968	Garcia Closas (2013)
rs4666451	2	19286943	G/A	0.39	0.42	0.97	0.94-1	0.92	0.83-1.01	0.093	0.84	0.071	0.93	0.234	Easton (2007)
rs12710696	2	19320803	C/T	0.31	0.36	1.1	1.06-1.13	1.12	1.01-1.25	0.034	1.2	0.062	1.13	0.070	Garcia Closas (2013)
rs4849887	2	121245122	C/T	0.12	0.098	0.9	0.84-0.96	0.9	0.78-1.05	0.198	1.02	0.863	0.91	0.329	Michailidou K (2013)
rs2016394	2	172972971	G/A	0.42	0.48	0.95	0.92-0.99	0.88	0.79-0.98	0.019	0.91	0.351	0.88	0.045	Michailidou K (2013)
rs1550623	2	174212894	A/G	0.23	0.16	0.91	0.86-0.96	0.92	0.82-1.04	0.170	0.81	0.069	1	0.999	Michailidou K (2013)
rs13393577	2	213296863	T/C	0.10	0.05	1.53	1.37-1.70	1.05	0.89-1.23	0.585	1.29	0.068	1.05	0.620	Kim HC (2012)
rs13387042	2	217905832	G/A	0.39	0.5	1.2	1.14-1.26	1.1	0.99-1.22	0.072	1.01	0.884	1.2	0.004	Stacey (2007)
rs16857609	2	218296508	C/T	0.38	0.26	1.09	1.05-1.14	0.94	0.85-1.04	0.202	1.01	0.890	0.9	0.115	Michailidou K (2013)
rs6762644	3	4742276	A/G	0.25	0.4	1.06	1.02-1.11	1	0.89-1.13	0.960	1.23	0.047	0.95	0.457	Michailidou K (2013)
rs4973768	3	27416013	C/T	0.57	0.47	1.16	1.1-1.24	1.04	0.94-1.15	0.418	0.97	0.709	1.06	0.354	Turnbull (2010)
rs12493607	3	30682939	G/C	0.33	0.35	1.04	1-1.09	1.03	0.93-1.15	0.580	0.93	0.486	1.04	0.559	Michailidou K (2013)
rs12505080	4	37349340	C/T	0.54	0.72	1.22	1.02-1.45	1	0.9-1.11	0.992	1	0.970	0.99	0.900	Hunter (2007)
rs9790517	4	106084778	C/T	0.31	0.23	1.09	1.04-1.14	0.94	0.84-1.05	0.264	0.82	0.052	0.96	0.556	Michailidou K (2013)
rs7697216	4	175828036	C/T	0.19	0.13	0.89	0.83-0.94	0.89	0.78-1	0.059	0.87	0.251	0.92	0.274	Michailidou K (2013)
rs6828523	4	175846426	C/A	0.19	0.13	0.89	0.83-0.94	0.88	0.77-1	0.045	0.86	0.214	0.9	0.192	Michailidou K (2013)
rs10069690	5	1279790	C/T	0.21	0.26	1.18	1.13-1.25	1.1	0.95-1.27	0.201	1.08	0.559	1.14	0.127	Garcia Closas (2013)
rs4415084	5	44662515	C/T	0.49	0.40	1.16	1.10-1.21	1.11	1-1.22	0.049	1.04	0.667	1.15	0.030	Stacey (2008)
rs10941679	5	44706498	A/G	0.34	0.25	1.13	1.1-1.15	1.12	1.01-1.25	0.033	1.13	0.211	1.17	0.022	Michailidou K (2013)
rs16886165	5	56023083	T/G	0.16	0.15	1.23	1.12-1.35	1.11	0.97-1.26	0.141	1.04	0.732	1.19	0.035	Thomas G. (2009)
rs889312	5	56031884	A/C	0.41	0.28	1.15	1.07-1.23	1.12	1.01-1.23	0.032	1.2	0.043	1.11	0.103	Easton (2007)
rs10472076	5	58184061	T/C	0.32	0.38	1.06	1.02-1.11	0.99	0.88-1.1	0.783	0.9	0.290	1.01	0.884	Michailidou K (2013)
rs1353747	5	58337481	T/G	0.09	0.095	0.9	0.84-0.96	1.04	0.88-1.23	0.675	0.86	0.375	1.07	0.531	Michailidou K (2013)
rs1432679	5	158244083	T/C	0.54	0.43	1.06	1.02-1.1	1.07	0.97-1.18	0.166	1.14	0.153	1.06	0.339	Michailidou K (2013)
rs11242675	6	1318878	T/C	0.45	0.39	0.97	0.93-1.01	0.92	0.83-1.01	0.094	0.87	0.135	0.93	0.257	Michailidou K (2013)
rs204247	6	13722523	A/G	0.42	0.43	1.06	1.02-1.1	0.99	0.89-1.09	0.800	0.91	0.286	1.02	0.773	Michailidou K (2013)
rs17530068	6	82193109	T/C	0.25	0.24	1.16	1.1-1.23	1.22	1.09-1.36	0.001	1.21	0.075	1.24	0.002	Siddiq (2012)+
rs2180341	6	127600630	A/G	0.24	0.21	1.41	1.25-1.59	1	0.89-1.12	0.997	0.93	0.482	0.95	0.462	Gold B (2008)
rs9485372	6	149608874	A/G	0.83	0.82	1.11	1.1-1.15	1.03	0.91-1.17	0.643	1.02	0.845	1.05	0.578	Long (2012)
rs3757318	6	151914113	G/A	0.06	0.07	1.13	1.17-1.46	1.08	0.87-1.33	0.483	1	0.991	1.1	0.453	Turnbull (2010)
rs2046210	6	151948366	G/A	0.28	0.37	1.29	1.21-1.37	1.06	0.95-1.19	0.276	1	0.974	1.08	0.266	Zheng (2009)
rs9383938	6	151987357	G/T	0.06	0.08	1.28	1.16-1.38	1.20	1.01-1.43	0.034	1.43	0.017	1.13	0.241	Siddiq (2012)+
rs720475	7	144074929	G/A	0.16	0.25	0.93	0.89-0.98	1.06	0.93-1.22	0.375	1.18	0.179	1.03	0.757	Michailidou K (2013)
rs9693444	8	29509616	C/A	0.33	0.32	1.07	1.03-1.12	1.01	0.91-1.13	0.808	0.99	0.892	0.99	0.890	Michailidou K (2013)
rs6472903	8	76230301	T/G	0.09	0.18	0.88	0.84-0.93	0.98	0.81-1.17	0.806	0.79	0.198	1.07	0.574	Michailidou K (2013)
rs2943559	8	76417937	A/G	0.08	0.07	1.17	1.09-1.26	1.15	0.96-1.39	0.126	1.19	0.286	1.12	0.304	Michailidou K (2013)
rs13281615	8	128355618	A/G	0.6	0.4	1.08	1.05-1.11	1.08	0.97-1.19	0.151	1.1	0.291	1.08	0.244	Easton (2007)
rs11780156	8	129194641	C/T	0.18	0.16	1.13	1.07-1.19	0.98	0.86-1.11	0.743	1.06	0.623	0.92	0.293	Michailidou K (2013)
rs1011970	9	22062134	G/T	0.35	0.17	1.09	1.04-1.14	1	0.9-1.11	0.998	1.16	0.122	0.87	0.036	Turnbull (2010)
rs10759243	9	110306115	C/A	0.43	0.39	1.07	1.02-1.12	1.12	1.02-1.24	0.024	1.14	0.150	1.12	0.076	Michailidou K (2013)

Chr.: Chromosomes; Pub.: Previously published; Lat.: Latinas; ER-neg: Estrogen receptor negative; ER-pos: Estrogen receptor positive

[*] Reference/tested

[^] Tested

[+] The samples included in the discovery phase of the present GWAS were included in the in silico replication stage of the Siddiq (2012) GWAS meta-analysis of ER-neg breast cancer

[#] SNPs were included in the analysis if they were registered in the Catalog of Published Genome-Wide Association Studies compiled by the National Human Genome Research Institute. In addition, we used a restriction criteria of p value lower than $5x10^{-8}$

Table 3: Breast cancer risk association in U.S Latinas for previously reported variants overall and by hormone receptor status CONT.

RS #	Chr.	Position	Alleles*	Freq. Latinas^	Freq. Pub^	OR Pub	95% CI Pub	OR Lat.	95% CI Lat.	P Lat.	OR ER-neg	P ER-neg	OR ER-pos	P ER-pos	Ref.#
rs865686	9	110888478	G/T	0.68	0.61	1.12	1.09-1.18	1.12	1.01-1.24	0.040	1.04	0.676	1.06	0.360	Fletcher (2011)
rs2380205	10	5886734	T/C	0.67	0.57	1.06	1.02-1.1	0.98	0.88-1.09	0.692	0.88	0.195	1.04	0.563	Turnbull (2010)
rs7072776	10	22032942	G/A	0.34	0.29	1.11	1.07-1.16	1.02	0.92-1.13	0.684	0.95	0.593	1.05	0.432	Michailidou K (2013)
rs11814448	10	22315843	A/C	0.06	0.02	1.35	1.17-1.56	1.16	0.94-1.44	0.158	1.1	0.630	1.28	0.057	Michailidou K (2013)
rs10822013	10	64251977	C/T	0.5	0.47	1.12	1.06-1.18	1.06	0.96-1.16	0.261	1.01	0.906	1.09	0.137	Cai (2011)
rs10995190	10	64278682	A/G	0.91	0.85	1.16	1.1-1.22	1.09	0.92-1.3	0.308	0.87	0.357	1.3	0.019	Turnbull (2010)
rs704010	10	80841148	C/T	0.42	0.39	1.07	1.03-1.11	1.13	1.02-1.25	0.022	1.09	0.366	1.11	0.088	Turnbull (2010)
rs11199914	10	123093901	C/T	0.44	0.32	0.94	0.89-0.98	1.01	0.92-1.11	0.848	1.02	0.835	1.03	0.679	Michailidou K (2013)
rs2981579	10	123337335	C/T	0.43	0.41	1.17	1.07-1.27	1.11	1-1.22	0.046	1.06	0.503	1.15	0.026	Thomas G. (2009)
rs1219648	10	123346190	A/G	0.41	0.4	1.2	1.07-1.42	1.12	1.01-1.24	0.026	1.09	0.334	1.16	0.017	Hunter (2007)
rs2981582	10	123352317	G/A	0.41	0.38	1.26	1.23-1.3	1.12	1.01-1.23	0.033	1.13	0.194	1.13	0.043	Easton (2007)
rs3817198	11	1909006	T/C	0.22	0.3	1.07	1.04-1.11	1.02	0.9-1.14	0.786	0.97	0.789	1	0.989	Easton (2007)
rs909116	11	1941946	C/T	0.42	0.53	1.17	1.1-1.24	1.08	0.97-1.19	0.174	0.97	0.757	1.09	0.175	Turnbull (2010)
rs2107425	11	2021075	C/T	0.47	0.29	0.86	0.81-0.92	0.9	0.81-0.99	0.039	0.91	0.329	0.86	0.013	Easton (2007)
rs3903072	11	65583066	G/T	0.33	0.47	0.92	0.89-0.96	1	0.9-1.12	0.940	0.94	0.555	1.02	0.789	Michailidou K (2013)
rs614367	11	69328764	C/T	0.08	0.15	1.15	1.1-1.2	1.08	0.9-1.29	0.398	0.75	0.134	1.19	0.103	Turnbull (2010)
rs11820646	11	129461171	C/T	0.45	0.41	0.93	0.9-0.97	0.94	0.85-1.03	0.186	0.95	0.537	0.9	0.093	Michailidou K (2013)
rs12422552	12	14413931	G/C	0.22	0.26	1.11	1.05-1.16	1.16	1.03-1.31	0.018	1.29	0.020	1.11	0.188	Michailidou K (2013)
rs10771399	12	28155080	C/T	0.92	0.9	1.2	1.15-1.27	1.1	0.92-1.32	0.310	1.28	0.171	0.96	0.726	Garcia Closas (2013)
rs17356907	12	96027759	A/G	0.37	0.3	0.89	0.85-0.93	0.99	0.89-1.09	0.819	0.96	0.664	0.95	0.440	Michailidou K (2013)
rs1292011	12	115836522	G/A	0.65	0.54	1.09	1.06-1.11	1.03	0.93-1.14	0.572	0.97	0.721	1.07	0.275	Michailidou K (2013)
rs2236007	14	37132769	G/A	0.12	0.21	0.88	0.83-0.93	0.94	0.8-1.09	0.405	1.1	0.490	0.92	0.403	Michailidou K (2013)
rs2588809	14	68660428	C/T	0.16	0.16	1.07	1.01-1.13	1.07	0.93-1.22	0.347	0.94	0.609	1.13	0.129	Michailidou K (2013)
rs999737	14	69034682	T/C	0.83	0.76	1.06	1.01-1.14	1.09	0.95-1.24	0.222	1.06	0.640	1.12	0.176	Thomas G. (2009)
rs941764	14	91841069	A/G	0.47	0.34	1.05	1-1.09	1.13	1.02-1.25	0.022	1.06	0.561	1.11	0.102	Michailidou K (2013)
rs3803662	16	52586341	C/T	0.39	0.27	1.28	1.21-1.35	1.31	1.19-1.45	$1.4x10^{-7}$	1.1	0.293	1.39	9.4E-08	Stacey (2007)
rs4784227	16	52599188	C/T	0.31	0.24	1.24	1.2-1.29	1.38	1.24-1.54	$3.0x10^{-9}$	1.2	0.070	1.43	5.1E-08	Long (2010)
rs17817449	16	53813367	T/G	0.27	0.4	0.95	0.91-0.99	0.97	0.87-1.08	0.580	0.88	0.232	1.01	0.939	Michailidou K (2013)
rs11075995	16	53855291	T/A	0.34	0.24	1.11	1.07-1.15	1.16	1.05-1.29	0.005	1.39	3.8E-04	1.05	0.467	Garcia Closas (2013)
rs13329835	16	80650805	A/G	0.19	0.22	1.14	1.09-1.19	1.12	0.99-1.27	0.074	1.1	0.419	1.11	0.188	Michailidou K (2013)
rs6504950	17	53056471	A/G	0.81	0.72	1.06	1.04-1.09	1.02	0.9-1.16	0.724	1.01	0.907	0.98	0.775	Michailidou K (2013)
rs527616	18	24337424	G/C	0.22	0.38	0.91	0.87-0.95	0.98	0.87-1.11	0.783	0.98	0.839	1	0.965	Michailidou K (2013)
rs1436904	18	24570667	T/G	0.44	0.4	0.93	0.9-0.97	0.95	0.85-1.05	0.278	0.97	0.784	0.94	0.314	Michailidou K (2013)
rs8170	19	17389704	G/A	0.11	0.17	1.26	1.17-1.35	1.03	0.88-1.21	0.716	1.37	0.021	0.94	0.534	Antoniou (2010)
rs2363956	19	17394124	A/C	0.42	0.57	0.8	0.74-0.87	0.99	0.89-1.09	0.790	0.85	0.091	1.02	0.808	Antoniou (2010)
rs4808801	19	18571141	A/G	0.38	0.35	0.94	0.9-0.98	0.88	0.79-0.97	0.012	0.8	0.021	0.88	0.044	Michailidou K (2013)
rs3760982	19	44286513	G/A	0.33	0.46	1.06	1.02-1.1	1.02	0.91-1.13	0.776	1.04	0.683	0.96	0.585	Michailidou K (2013)
rs2284378	20	32588095	C/T	0.31	0.31	1.16	1.1-1.22	1.23	1.11-1.37	$1.3x10^{-4}$	1.38	0.001	1.23	0.002	Siddiq (2012)+
rs2823093	21	16520832	A/G	0.71	0.73	1.09	1.06-1.11	1.1	0.98-1.23	0.102	0.94	0.541	1.16	0.040	Michailidou K (2013)
rs6001930	22	40876234	T/C	0.09	0.11	1.17	1.11-1.25	1.16	0.98-1.38	0.089	0.84	0.320	1.19	0.106	Michailidou K (2013)

Chr.: Chromosomes; Pub.: Previously published; Lat.: Latinas; ER-neg: Estrogen receptor negative; ER-pos: Estrogen receptor positive

*Reference/tested

^Tested

+The samples included in the discovery phase of the present GWAS were included in the in silico replication stage of the Siddiq (2012) GWAS meta-analysis of ER-neg breast cancer

#SNPs were included in the analysis if they were registered in the Catalog of Published Genome-Wide Association Studies compiled by the National Human Genome Research Institute. In addition, we used a restriction criteria of p value lower than $5x10^{-8}$.

Table 4: SNPs showing strongest association from the Multiethnic Cohort (MEC) sequenced data (451 cases and 456 controls) and odds ratios and p values for GWAS results including all samples in the discovery phase

CHR	SNP	BP	A1	A2	Freq A1	OR-Seq	P-Seq	OR-GWAS	P-GWAS[d]
6	rs4524618[a]	151918023	A	G	0.13	0.55	0.0001	0.70	1.23×10^{-5}
6	rs9397432[b]	151916855	C	G	0.12	0.59	0.0011	0.70	1.25×10^{-5}
6	rs79692348[c]	151937679	T	C	0.13	0.63	0.0023	0.69	7.93×10^{-6}
**6**	**rs140068132**	**151954834**	**G**	**A**	**0.09**	**0.60**	**0.0035**	**0.59**	**2.41×10^{-7}**
**6**	**rs147157845**	**151971376**	**A**	**C**	**0.09**	**0.63**	**0.0074**	**0.58**	**7.44×10^{-8}**

[a] r^2 of 0.55 with rs140068132
[b] r^2 of 0.58 with rs140068132
[c] r^2 of 0.64 with rs140068132
[d] We conducted a logistic regression analysis for rs140068132 including the principal components 1-10, rs4524618, rs9397432 and rs79692348 as covariates in all the discovery phase samples and did not observed any independent effect of variants rs4524618, rs9397432 and rs79692348 on breast cancer risk beyond that of rs140068132.

Table 5: Association between rs140068132/rs147157845 and breast cancer risk by ER status in subset of samples from the Colombia breast cancer study.

**ER status (131 ER+, 87 ER-, 440 controls)**	**OR[a]**	**95% CI**	**P value**
**rs140068132**			
**ER+**	0.62	0.40-0.96	0.029
**ER-**	0.48	0.27-0.84	$9.5x10^{-3}$
**ER- vs. ER+**	0.77	0.40-1.49	0.444
**rs147157845**			
**ER+**	0.62	0.40-0.96	0.031
**ER-**	0.52	0.30-0.90	0.019
**ER- vs. ER+**	0.84	0.44-1.60	0.591

ER: Estrogen Receptor; ER+: Estrogen Receptor Positive; ER-: Estrogen Receptor Negative
[a]ORs are for the minor alleles (rs140068132 allele G & rs147157845 allele A)

Table 6: Association between rs140068132/rs147157845 and percent mammographic density in Mexican women

6.a) Linear regression: Effect of rs140068132 polymorphism on mammographic density among controls (N=809)

	**Coef.**	**Std. Err.**	**t**	**P>|t|**	**L95%CI**	**H95%CI**
**rs140068132**	**-0.160**	**0.047**	**-3.390**	**0.001**	**-0.253**	**-0.068**
Ind.Am. Ancestry	-0.011	0.146	-0.070	0.943	-0.297	0.276
African Ancestry	0.869	0.499	1.740	0.082	-0.111	1.849
Age	-0.025	0.003	-7.430	<0.001	-0.032	-0.018
Body Mass Index	-0.049	0.005	-10.090	<0.001	-0.059	-0.040
Height	-0.056	0.012	-4.510	<0.001	-0.081	-0.032
Parity	-0.067	0.076	-0.870	0.383	-0.217	0.083
Breast feeding	0.008	0.005	1.710	0.087	-0.001	0.017

6.b) Linear regression: Effect of rs147157845 polymorphism on mammographic density among controls (N=809)

**logDM**	**Coef.**	**Std. Err.**	**t**	**P>|t|**	**L95%CI**	**H95%CI**
**rs147157845**	**-0.133**	**0.048**	**-2.790**	**0.005**	**-0.226**	**-0.039**
Ind.Am. Ancestry	-0.028	0.146	-0.190	0.847	-0.315	0.259
African Ancestry	0.861	0.500	1.720	0.085	-0.120	1.843
Age	-0.025	0.003	-7.370	<0.001	-0.032	-0.018
Body Mass Index	-0.049	0.005	-10.070	<0.001	-0.059	-0.040
Height	-0.056	0.012	-4.450	<0.001	-0.080	-0.031
Parity	-0.071	0.077	-0.920	0.357	-0.221	0.080
Breast feeding	0.008	0.005	1.740	0.082	-0.001	0.017

6.c) Logistic regression: case vs. control status adjusting for covariates but without density (Overall effect of rs140068132 on breast cancer risk), N=1113 (304 cases & 809 controls)

	**Coef.**	**Std. Err.**	**z**	**P>|z|**	**L95%CI**	**H95%CI**
**rs140068132**	**-0.310**	**0.146**	**-2.130**	**0.034**	**-0.597**	**-0.024**
Ind.Am. Ancestry	-1.087	0.394	-2.760	0.006	-1.859	-0.315
African Ancestry	0.028	1.299	0.020	0.983	-2.517	2.573
Age	0.040	0.009	4.640	<0.001	0.023	0.057
Body Mass Index	-0.047	0.014	-3.340	0.001	-0.075	-0.019
Height	0.026	0.012	2.080	0.038	0.001	0.050
Parity	-0.144	0.036	-3.960	<0.001	-0.215	-0.073
Breast feeding	-0.268	0.192	-1.390	0.163	-0.644	0.109

6.d) Logistic regression: case vs. control status adjusting for covariates including density (Direct effect of rs140068132 on breast cancer risk)

	**Coef.**	**Std. Err.**	**z**	**P>|z|**	**L95%CI**	**H95%CI**
**rs140068132**	**-0.262**	**0.148**	**-1.760**	**0.078**	**-0.552**	**0.029**
Ind.Am. Ancestry	-1.080	0.396	-2.730	0.006	-1.857	-0.304
African Ancestry	-0.213	1.312	-0.160	0.871	-2.785	2.360
Age	0.051	0.009	5.570	<0.001	0.033	0.069
Body Mass Index	-0.028	0.015	-1.910	0.056	-0.058	0.001
Height	0.023	0.012	1.850	0.065	-0.001	0.046
Parity	-0.127	0.037	-3.450	0.001	-0.199	-0.055
Breast feeding	-0.256	0.194	-1.320	0.186	-0.636	0.124
Log Mammographic density	0.391	0.098	4.000	<0.001	0.199	0.583

Figure 1: Correlation (r^2) between previously reported risk variants at 6q25 (bold red), rs140068132/rs147157845 (bold black) and other SNPs in the region with P values <0.05 in the sequence data breast cancer case/control analysis among the U.S. Latinas from the Multiethnic Cohort (black). r2 categories are <0.2 (lighter blue), 0.2-0.4 (turquoise), and >0.4 (darker blue).

SNP	rs4869732	rs1482061	rs143390228	rs75318086	rs9383580	rs9397426	**rs3757318**	rs9397432	rs4524618	**rs12662670**	**rs6929137**	rs79692348	**rs6913578**	**rs9397435**	**rs140068132**	**rs147157845**	**rs10484919**	rs78841457	rs78177662	rs59623026	rs17081551	rs58374672	rs77315705	rs77758681
Position	151822015	151823813	151835532	151839433	151855743	151874558	**151914113**	151916855	151918023	**151918856**	**151936677**	151937679	**151949806**	**151951220**	**151954834**	**151971376**	**151974422**	151974492	151975565	151978964	151980730	151982681	151982879	151983383
rs1482061	0.988																							
rs143390228	0.994	0.983																						
rs75318086	0.983	0.994	0.988																					
rs9383580	0.496	0.489	0.500	0.494																				
rs9397426	0.582	0.574	0.588	0.580	0.780																			
**rs3757318**	0.007	0.007	0.007	0.007	0.008	0.009																		
rs9397432	0.229	0.225	0.233	0.228	0.326	0.347	0.009																	
rs4524618	0.232	0.228	0.235	0.231	0.326	0.339	0.012	0.983																
**rs12662670**	0.005	0.005	0.005	0.006	0.009	0.009	0.828	0.008	0.011															
**rs6929137**	0.027	0.025	0.027	0.026	0.029	0.027	0.153	0.040	0.040	0.159														
rs79692348	0.222	0.218	0.225	0.221	0.304	0.320	0.012	0.845	0.833	0.012	0.038													
**rs6913578**	0.025	0.026	0.026	0.027	0.020	0.024	0.083	0.049	0.045	0.099	0.753	0.042												
**rs9397435**	0.004	0.004	0.004	0.005	0.007	0.009	0.527	0.010	0.013	0.574	0.180	0.014	0.186											
**rs140068132**	0.294	0.290	0.299	0.294	0.354	0.426	0.009	0.575	0.552	0.008	0.025	0.637	0.029	0.007										
**rs147157845**	0.287	0.282	0.291	0.287	0.338	0.407	0.009	0.557	0.536	0.007	0.023	0.618	0.027	0.006	0.974									
**rs10484919**	0.005	0.006	0.005	0.006	0.008	0.008	0.286	0.011	0.010	0.345	0.116	0.011	0.152	0.539	0.008	0.008								
rs78841457	0.202	0.198	0.205	0.201	0.272	0.291	0.016	0.770	0.760	0.012	0.033	0.855	0.035	0.016	0.635	0.641	0.014							
rs78177662	0.212	0.208	0.215	0.211	0.255	0.275	0.017	0.735	0.714	0.014	0.037	0.806	0.039	0.017	0.603	0.609	0.016	0.941						
rs59623026	0.210	0.206	0.213	0.209	0.253	0.273	0.017	0.740	0.719	0.014	0.038	0.802	0.040	0.017	0.600	0.606	0.016	0.936	0.996					
rs17081551	0.211	0.207	0.213	0.209	0.253	0.273	0.017	0.740	0.719	0.014	0.037	0.802	0.039	0.017	0.600	0.606	0.016	0.937	0.996	1.000				
rs58374672	0.212	0.208	0.215	0.211	0.255	0.275	0.017	0.735	0.714	0.014	0.037	0.806	0.039	0.017	0.603	0.609	0.016	0.941	1.000	0.996	0.996			
rs77315705	0.197	0.193	0.200	0.196	0.237	0.262	0.015	0.712	0.689	0.011	0.036	0.794	0.037	0.016	0.592	0.594	0.015	0.919	0.982	0.977	0.977	0.982		
rs77758681	0.212	0.208	0.215	0.211	0.255	0.275	0.017	0.735	0.714	0.014	0.037	0.806	0.039	0.017	0.603	0.609	0.016	0.941	1.000	0.996	0.996	1.000	0.982	
rs7756917	0.073	0.072	0.073	0.073	0.121	0.112	0.023	0.206	0.197	0.026	0.003	0.232	0.005	0.051	0.260	0.257	0.072	0.225	0.207	0.205	0.206	0.207	0.203	0.207

Figure 2: Scatterplot of tested allele frequencies previously published (X axis) and in Latinas (Y axis)

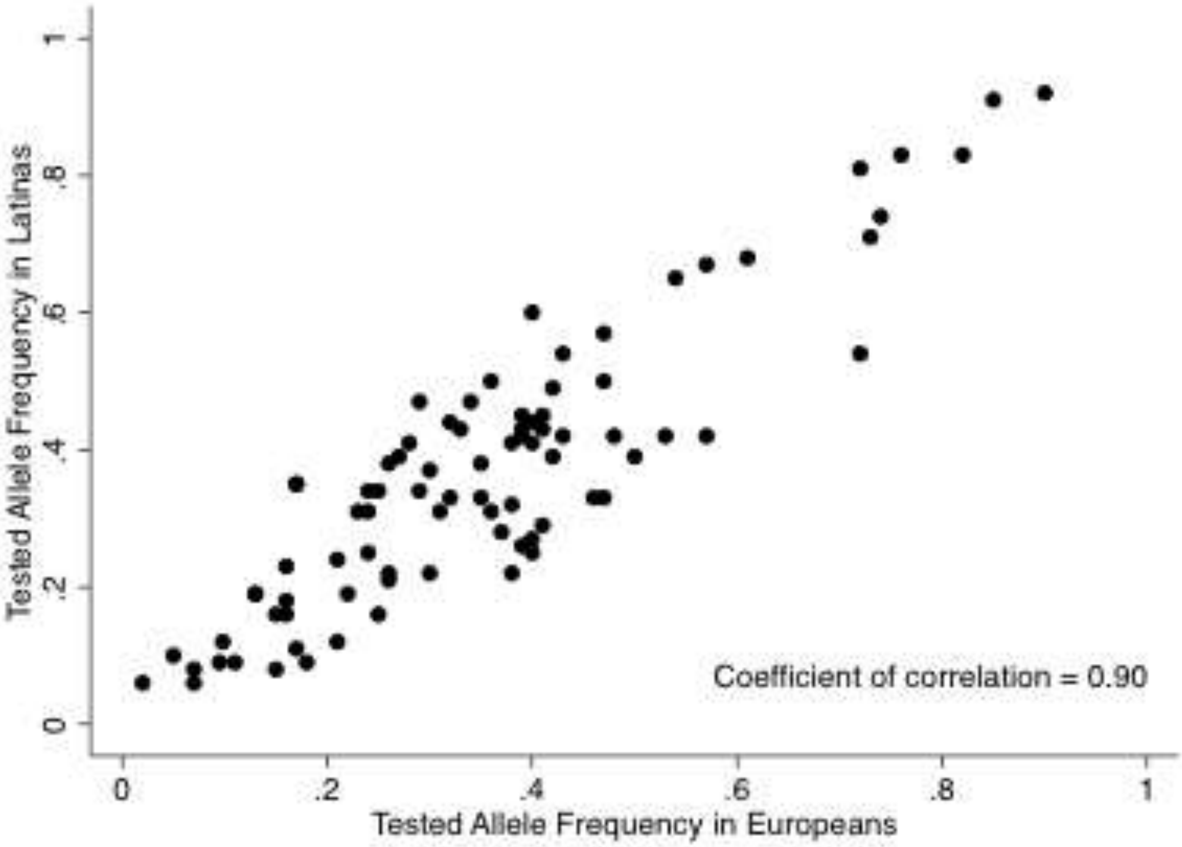

Figure 3: UCSC genome browser with ENCODE transcription factor binding sites track (the location of rs140068132 and rs147157845 is included within the figure)

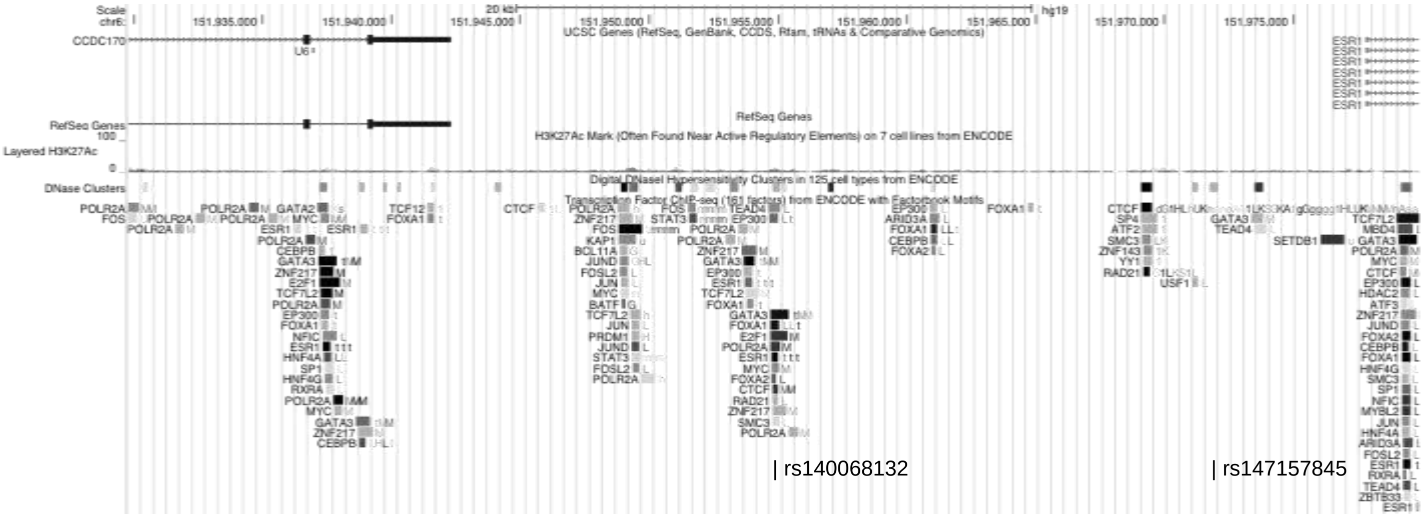

Figure 4: EMSA demonstrating control reaction lanes 1-3 and differential binding of A/G SNP rs140068132 to nuclear protein derived from MCF-7 cells, A allele lanes 4-6, G allele lanes 7-9. Shift is demonstrated in lanes 5 and 8, respectively, with greater protein binding in lane 5 with A allele as compared to lane 8 with G allele. This shift is specific to the sequence including the SNP as a 500 fold molar excess of unbiotinylated primers of identical sequence quenches the reactions as demonstrated in lanes 6 and 9.

Lane	1	2	3	4	5	6	7	8	9
Unbiotinylated Primer	-	-	+	-	-	+	-	-	+
Nuclear Protein Extract	-	+	+	-	+	+	-	+	+
Biotinylated Primer	+	+	+	+	+	+	+	+	+

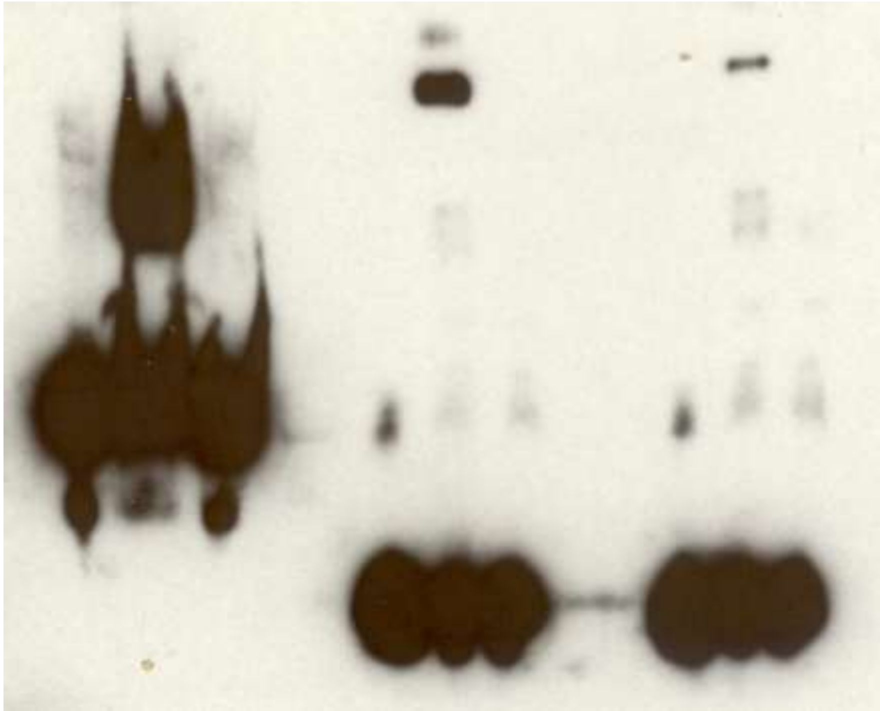

Figure 5: Scatter plot of 1st and 2nd principal components among U.S. Latinas included in the discovery phase of the breast cancer GWAS (N=4710) and HapMap ancestral samples

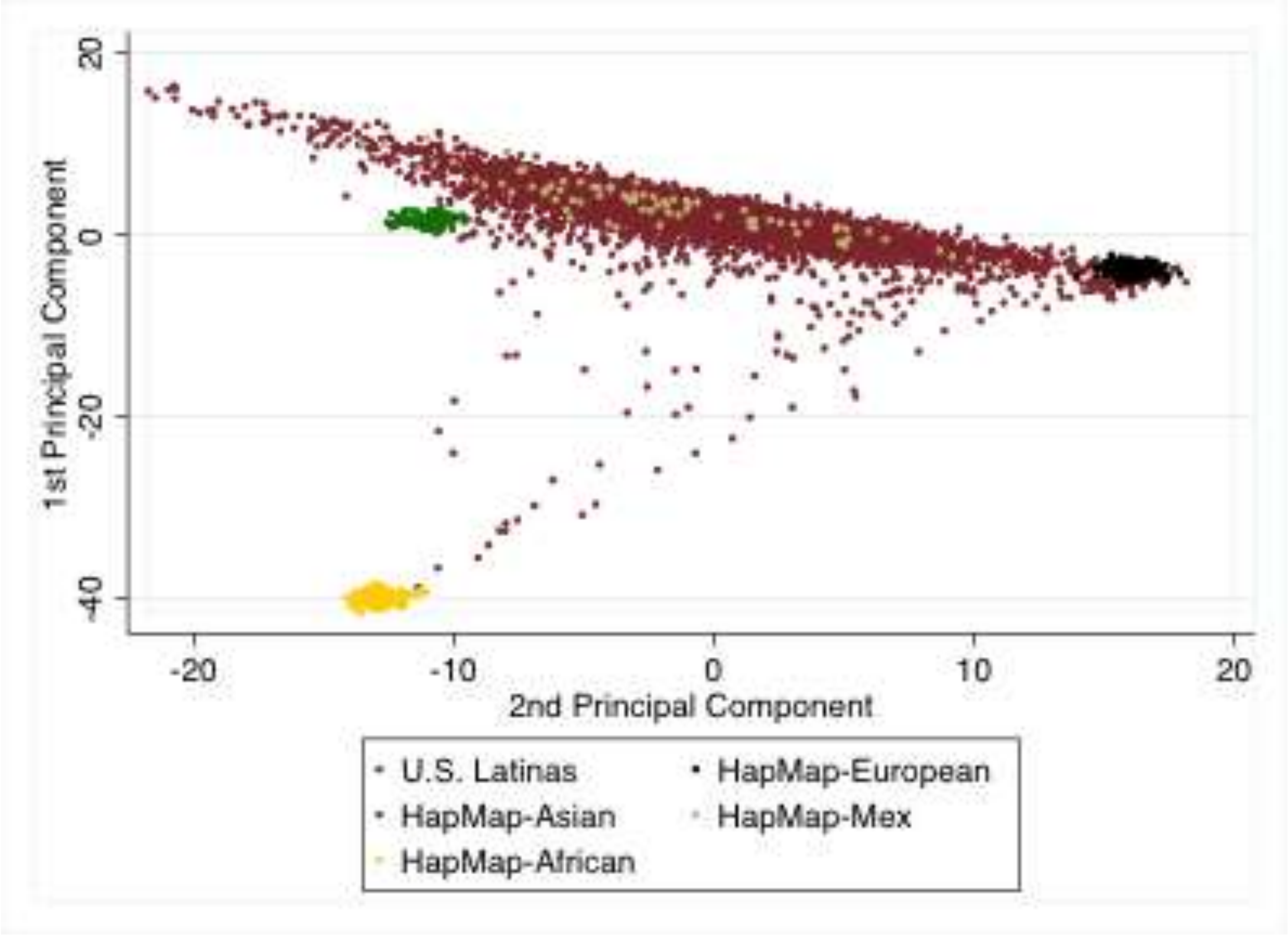

Supplement: Supplementary Information — Supplementary Figures 1-5 and Supplementary Tables 1-6 [file ncomms6260-s1.pdf]
